# Computational Screening of Natural Compounds for Identification of Potential Anti-Cancer Agents Targeting MCM7 Protein

**DOI:** 10.3390/molecules26195878

**Published:** 2021-09-28

**Authors:** Mohammad Y. Alshahrani, Kholoud M. Alshahrani, Munazzah Tasleem, Arshiya Akeel, Tahani M. Almeleebia, Irfan Ahmad, Mohammed Asiri, Najla A. Alshahrani, Nadiyah M. Alabdallah, Mohd Saeed

**Affiliations:** 1 Department of Clinical Laboratory Sciences, College of Applied Medical Sciences, King Khalid University, P.O. Box 61413, Abha 62529, Saudi Arabia; moyahya@kku.edu.sa (M.Y.A.); irfancsmmu@gmail.com (I.A.); masiri@kku.edu.sa (M.A.); 2 College of Medicine, King Khalid University Abha, P.O. Box 61413, Abha 62529, Saudi Arabia; Kholoudalbjadi@gmail.com (K.M.A.); aabshahrani@gmail.com (N.A.A.); 3 School of Electronic Science and Engineering, University of Electronic Science and Technology of China, Chengdu 610054, China; munazzah.t@gmail.com; 4 Department of Botany, Aligarh Muslim University, Aligarh 202002, India; 5 Department of Clinical Pharmacy, College of Pharmacy, King Khalid University, P.O. Box 61413, Abha 62529, Saudi Arabia; talmelby@kku.edu.sa; 6 Department of Biology, College of Science, Imam Abdulrahman Bin Faisal University, P.O. Box 1982, Dammam 31441, Saudi Arabia; nmalabdallah@iau.edu.sa; 7 Department of Biology, College of Sciences, University of Hail, P.O. Box 2440, Hail 55425, Saudi Arabia

**Keywords:** cancer, DNA synthesis, MCM7, natural products

## Abstract

Minichromosome maintenance complex component 7 (MCM7) is involved in replicative licensing and the synthesis of DNA, and its overexpression is a fascinating biomarker for various cancer types. There is currently no effective agent that can prevent the development of cancer caused by the MCM7 protein. However, on the molecular level, inhibiting MCM7 lowers cancer-related cellular growth. With this purpose, this study screened 452 biogenic compounds extracted from the UEFS Natural Products dataset against MCM protein by using the in silico art of technique. The hit compounds UEFS99, UEFS137, and UEFS428 showed good binding with the MCM7 protein with binding energy values of −9.95, −8.92, and −8.71 kcal/mol, which was comparatively higher than that of the control compound ciprofloxacin (−6.50). The hit (UEFS99) with the minimum binding energy was picked for molecular dynamics (MD) simulation investigation, and it demonstrated stability at 30 ns. Computational prediction of physicochemical property evaluation revealed that these hits are non-toxic and have good drug-likeness features. It is suggested that hit compounds UEFS99, UEFS137, and UEFS428 pave the way for further bench work validation in novel inhibitor development against MCM7 to fight the cancers.

## 1. Introduction

Cancer chemotherapy has historically targeted DNA replication because cancer cells proliferate uncontrollably compared to most non-cancerous cells [1]. The Mcm2-7 helicase (minichromosome maintenance protein 2–7), a well-known molecular motor that winds down duplex DNA to create ssDNA templates for replication, is one potential therapeutic target [2]. Small molecule inhibitors that primarily target the leading or lagging strand of DNA synthesis were employed earlier to clinically block the proliferation of uncontrolled cancer cells [3]. Although these compounds were shown to be chemotherapeutic, they are aimed at both normal and malignant DNA replication and frequently have adverse effects. Therefore, few inhibitors that target replication initiation were developed [4,5]. 

The toroidal Mcm2-7 complex, unlike other replicative helicases, is composed of six distinct and essential subunits (numbered 2–7) [2]. Each subunit is an AAA+ ATPase, and the helicase’s distinct hetero-hexameric composition was conserved throughout eukaryotic evolution [2]. The fact that certain mutations in Mcm2-7 and overexpression of its subunits induce cancer or contribute to tumor formation highlights the necessity of its control [6]. Even though helicases have the potential to be disease targets, there are just a few particular small molecule antagonists of these enzymes [7,8]. So far, heliquinomycin was identified as an inhibitor of a non-physiological Mcm sub complex (Mcm467) [9,10] and reduces cancer cell proliferation in vitro, implying that Mcm inhibitors possess therapeutic potential [11]. Furthermore, enzyme inhibitors have a long and distinguished history in biochemical research, and their use has proven to be a challenging route for gaining critical mechanistic insights [12]. Due to the shortcomings of existing clinical trials, phytochemical compounds are intensely focused on treating different cancers [13].

Over the last several decades, improvements in anticancer drug research and development have resulted in the discovery and approval of more than 100 anticancer medicines by the FDA [14,15]. Based on their mode of action, these drugs can be divided into two main categories: cytotoxic and targeted agents [16,17]. Cytotoxic drugs can kill rapidly dividing cells by targeting components of the mitotic and/or DNA replication processes. The targeted drugs inhibit cancer development and spread by interacting with molecular targets implicated in cancer growth, progression, and dissemination pathways [18]. Those effective medicines, as well as their associated data, may give useful information for future therapeutic target identification, anticancer drug combination development, drug repurposing, and computational pharmacology.

There are only limited treatment options for cancer, including chemotherapy, radiation, and surgical removal. However, these treatment methods are less successful because of recurring cancer, chemical resistance, and the effect of non-targeted cells. Furthermore, many antitumor agents are plagued by issues of rather undesirable side effects. This affirms the impediment of cytotoxic chemotherapy. Thus, new agents to extend cancer prevention chemotherapy remain vital to be discovered [19].

Drug development necessitates the selection of a few potential hits from a vast library of chemical components. Although, it can be challenging to screen such an extensive compound library using wet-lab assays [20]. Molecular docking is an influential tool for drug development because it helps identify active or lead compounds in a compound library. Computational screening before laboratory testing is a successful approach to decreasing candidate inhibitors for benchwork-based screening [21]. The present study aimed to identify new possible hits from the natural compounds databases using in silico state-of-the-art techniques that could serve as Mcm2-7 inhibitors to fight against cancer.

## 2. Methodology

### 2.1. Protein Preparation

The 3D structure of the MCM7 protein retrieved from protein data bank [PDB ID: 6XTX] is a hetero 11-mer. The chain ‘F’ is the MCM7 having 719 amino acids [22]. All the chains except ‘F’ and cofactors of the protein were deleted, and the protein was prepared using Discovery Studio 2020 and saved separately for the screening/docking analysis.

### 2.2. Compound Library Preparation 

Natural compounds were accessed from the ZINC database under the catalogue “UEFS Natural Products” which is a collection of biogenic compounds with an “In-Vitro” activity level [23]. 452 biogenic compounds were extracted from the UEFS Natural Products dataset and refined in Discover Studio 2020 using the ligand preparation tool.

### 2.3. Receptor-Based Virtual Screening 

Using the AutoDock Vina program in PyRx software, the prepared library of biogenic compounds was computationally screened against the catalytic site of MCM7 protein [24]. The SDF format of the compound library was imported into PyRx workspace and processed for minimization. Then, using Open Babel in PyRx program, these minimized ligands were translated into pdbqt format. Lastly, the top-scoring compounds were subjected to a more comprehensive docking analysis.

### 2.4. Molecular Docking

The best-screened hits were further docked to the MCM7 protein active site using the ‘Autodock4.2’. A semi-flexible docking technique was employed. The ligand was flexible while the protein was treated as a rigid molecule. The energy of docked ligands (hit compounds) was minimized using the MMFF94 force field. The Autogrid tool was used to create grid maps with specific coordinates for docking hit compounds into the active site of the MCM7 protein. Grid points were kept as 40 × 40 × 40 Å with 0.375 Å spacing. However, the X, Y, and Z coordinates were set as 212.0711, 231.253, and 164.717, respectively. Docking simulation was accomplished using the Solis & Wets local search and the Lamarckian genetic algorithm method. For each docking, hundreds of different runs were used with a total limit of 2,500,000 energy assessments. The binding pose with the highest negative binding energy (BE) value was deemed the most promising. 

### 2.5. Pharmacokinetics and Toxicity Prediction

SwissADME (http://www.swissadme.ch/, accessed on 15 April 2021), a web tool with competent in-house methods like iLOGP and the BOILED-Egg, was utilized to generate robust prediction models for physicochemical characteristics (Supplementary Figure S1), pharmacokinetics, and druglikeness of the top three screened compounds [25]. The ProTox-II webserver (https://tox-new.charite.de/protox_II/, accessed on 15 April 2021) was used to predict toxicity of the best three compounds. ProTox-II accepts a two-dimensional chemical structure/smile ID as input and produces a toxicity profile for 33 models with confidence scores, an overall toxicity radar graphic, and three most comparable compounds with documented acute toxicity [26].

### 2.6. Molecular Dynamics Simulations

GROMACS 2018.1 [27] was used to run a 30 ns molecular dynamics (MD) simulation with the CHARMM36 [28] all-atom force field. To explain the factors underlying one of the top-screened compounds (UEFS99) efficiency in inhibiting MCM7, MD simulations of the UEFS99-MCM7 complex was performed. The MCM7 structure, as well as the docked complex of MCM7and UEFS99, was immersed in the center of a dodecahedron box of a simple point charge (SPC) water model with a minimum distance of 1.0 nm between the wall and any component of the protein set up at the start of the simulation. To neutralize the solvated system, treatment with the aqueous environment of 0.1 M ionic strength was performed by adding Na^+^ (sodium) and Cl^−^ (chloride). After minimization, soft coupling with the modified berendsen thermostat (NVT) was applied to heat the system for 100 ps. Periodic boundary conditions (PBC) were applied with a constant number of particles in the system, constant pressure, and constant temperature simulation criteria (NPT) to carry out 30 ns simulations for MCM7 structure and complex MCM7 with UEFS99.

## 3. Result and Discussion

### 3.1. Virtual Screening and Molecular Docking

Computer-assisted drug design has become one of the essential techniques in modern drug design, as it can minimize the cost, time, and labour involved in the drug development process [29]. Since cancer cells proliferate uncontrollably compared to non-cancerous cells, DNA replication was long regarded as a key target for cancer chemotherapy. One potential therapeutic target is the MCM7 protein, a crucial component of the DNA replication-licensing complex and is over-expressed in various cancers [30,31,32].

**Figure 1:**
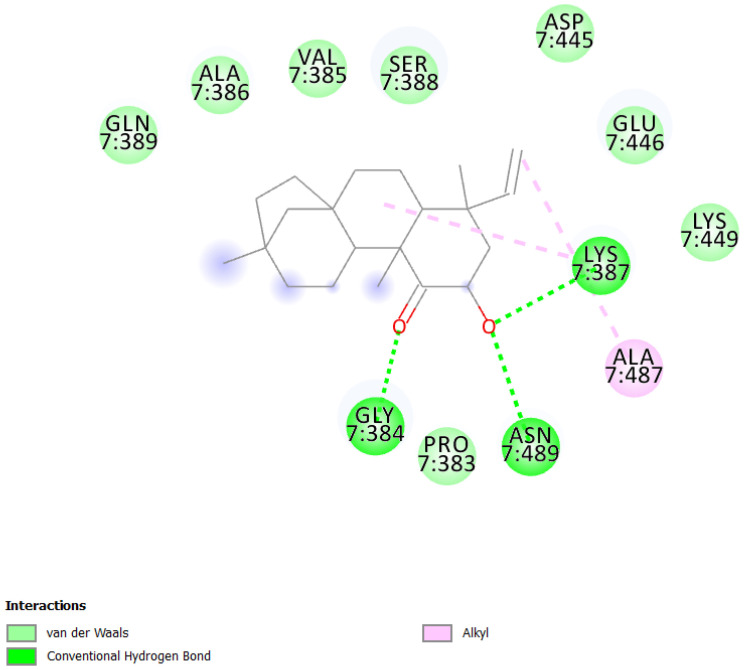
MCM7 protein-interacting residues with UEFS99. Different color code represent the residual interaction types.

**Figure 2:**
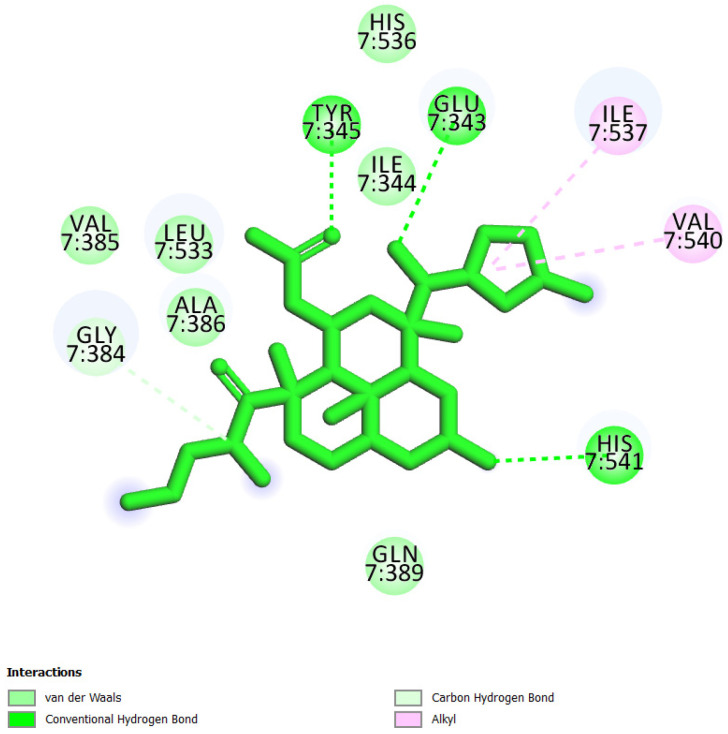
MCM7 protein-interacting residues with UEFS137. Different color codes represent the residual interaction types.

**Figure 3:**
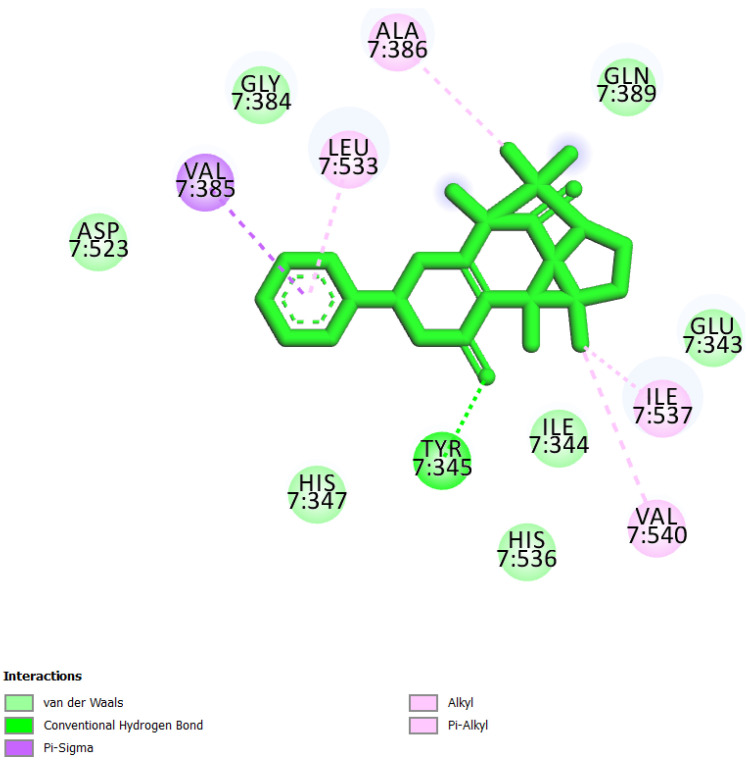
MCM7 protein-interacting residues with UEFS428. Different color code represents the residual interaction types.

In this study, we performed a computational screening of 452 biogenic compounds extracted from the UEFS Natural Products dataset to target MCM7 protein. Among them, the top three hits UEFS99, UEFS137, and UEFS428 showed good binding with the MCM7 protein. The docking analysis showed that 12 amino acid residues (Pro383, Gly384, Val385, Ala386, Lys387, Ser388, Gln389, Asp445, Glu446, Lys449, Ala487, and Asn489) of the MCM7active site were involved in interactions with UEFS99. Pro383, Val385, Ala386, Ser388, Gln389, Asp445, Glu446, and Lys449 residues of MCM7 were interacted with UEFS99 via van der Waals interaction (Figure 1). UEFS137 was found to interact with Glu343, Ile344, Tyr345, Gly384, Val385, Ala386, Gln389, Leu533, His536, Ile537, Val540, and His541 residues of MCM7 protein. UEFS137 form H-bond with Glu343, Ile344, and His541 residues of MCM7, while Ile537, and Val540 make the alkyl interaction (Figure 2). Further, Glu343, Ile344, Tyr345, His347, Gly384, Val385, Ala386, Gln389, Asp523, Leu533, His536, Ile537, and Val540 residues of MCM7 protein was observed to interact with UEFS428.Glu343, Ile344, His347, Gly384, Gln389, Asp523, and His536 residues were involved in van der Waals interaction with UEFS428, while Tyr345 make the H-bond (Figure 3).

BE and inhibition constant for ‘UEFS99-MCM7’, ‘UEFS137-MCM7’, and ‘UEFS428-MCM7’ complexes were found to be ‘−9.95 kcal/mol and 2.49 µM’, ‘−8.92 kcal/mol and 5.13 µM’, and ‘−8.71 kcal/mol and 9.53 µM’, respectively (Table 1). 

An enzyme’s active site is a region of the enzyme with a certain shape that causes it to associate with a specific substrate, causing it to undergo a chemical reaction [33]. The active site maintains optimal and desirable catalytic microenvironments and aids compounds in forming sufficient contact points to produce good binding with the target protein. MCM7 active site residues were demonstrated as Glu343, Ile344, Tyr345, Pro383, Gly384, Val385, Ala386, Lys387, Ser388, Gln389, Asn489, and Leu533 [34]. Interestingly, UEFS99, UEFS137, and UEFS428 were also observed to bind with these MCM7 protein residues.

**Figure 4:**
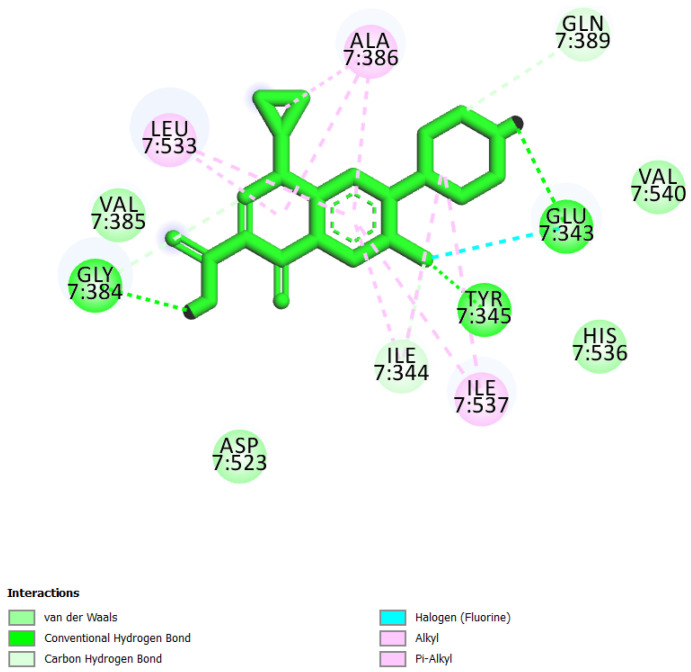
MCM7 protein-interacting residues with ciprofloxacin. Different color codes represent the residual interaction types.

Hydrogen bonds aid in determining the efficacy of the inhibitor against the target protein and play a key role in its stability with the enzyme/protein [35]. Tyr345 was the common H-bond interacting residue of MCM7 protein with ciprofloxacin, UEFS137, and UEFS428 (Figure 2, Figure 3 and Figure 4). In line with this, a recent study found that the Tyr345 residue of the MCM7 protein forms H-bonds with phytochemicals [34]. Despite the fact that UEFS99 was not interacted with Tyr345 via H-bond, it makes H-bond with several other residues (Gly384, Lys387, and Asn489) of MCM7 protein (Figure 1).

This study used ciprofloxacin as a reference ligand due to its previously documented inhibitory activity against MCM7 protein with an IC_50_ of 632 µM. Gly384, Val385, Ala386, and Gln389 were the common interacting residues of MCM7 protein with ciprofloxacin and the selected hit compounds (Figure 1, Figure 2, Figure 3 and Figure 4). The BE of ciprofloxacin with MCM7 protein was found to be −6.50 kcal/mol (Table 1).

Furthermore, to get a clearer picture of MCM7 protein-interacting residues with the hits, we analyzed MCM7 protein-interacting residues with its co-crystallized ligand: phosphothiophosphoric acid-adenylate ester(PDB ID:6XTX) [22], which revealed that Glu343, Ile344, Tyr345, Pro383, Gly384, Val385, Ala386, Lys387, Ser388, Gln389, Asn489, Leu533 and Ile537 are essential in binding with its co-crystallized ligand (Supplementary Figure S2). Interestingly, Gly384, Val385, and Ala386 are the common interacting MCM7 protein residues with the UEFS99, UEFS137, and UEFS428 as well as the co-crystalized ligand (Figure 1, Figure 2 and Figure 3 and Supplementary Figure S2).

Biogenic compounds and their derivatives are an excellent source of molecules to be investigated for anticancer activities. In cancer pharmaceutics, 49% of chemotherapy drugs are derived from or inspired by natural sources such as plants, microbes, and marine organisms [36]. Vinca alkaloids, taxanes (tubulin-binding drugs), podophyllotoxins, anthracyclines, and etoposides are a few examples [37,38]. Quinones were long a source of cytotoxic chemicals, found in various drugs such as the anthracyclines daunorubicin, doxorubicin, and mitoxantrone, which are used clinically in cancer therapy. Many additional pharmacological actions were documented, including antiallergic [39], antibacterial [40], anti-inflammatory [41], antithrombotic [42], and antiplatelet agents [39].

Chemotherapy, radiation therapy, and surgical removal are the only treatment options available for cancer patients; phytochemical compounds are considered an alternative therapeutic option to improve treatment efficiency. Additional issues, such as the side effects of standard treatments like chemotherapy [43,44] and radiation [45], increase the burden and make it more difficult to treat cancer patients. Molecular docking is a method of predicting the conformation of a ligand-receptor complex using a computer simulation, and it is becoming an increasingly valuable method of drug development [46]. BE determines the degree of interaction between ligand-protein complex, and a high (negative) value indicates that the inhibitor successfully binds to its target protein [47,48]. As a result, the detachment rate of such a ligand from its target protein is lesser, and this ligand is expected to have a longer half-life [49]. Accordingly, in the present study, the hits (UEFS99, UEFS137, and UEFS428) exhibited stronger binding (lower BE) to the MCM7 protein than the reference ligand (ciprofloxacin), implying that these compounds could inhibit the MCM7 protein and be used as a promising and efficient anticancer agent.

### 3.2. Physicochemical and Drug Likeness Properties

Several drug development failures can be attributed to inadequate pharmacokinetics and bioavailability, in addition to potency and toxicity. Two pharmacokinetic characteristics that should be estimated at various phases of the drug development process are gastrointestinal absorption and brain access [50]. The physicochemical characteristics, druglikeness, and toxicity evaluation of the top three compounds reveal that they have essentially all of the qualities needed to be future drug-like molecules (Table 2 and Table 3)**.**

### 3.3. MD Simulations

**Figure 5:**
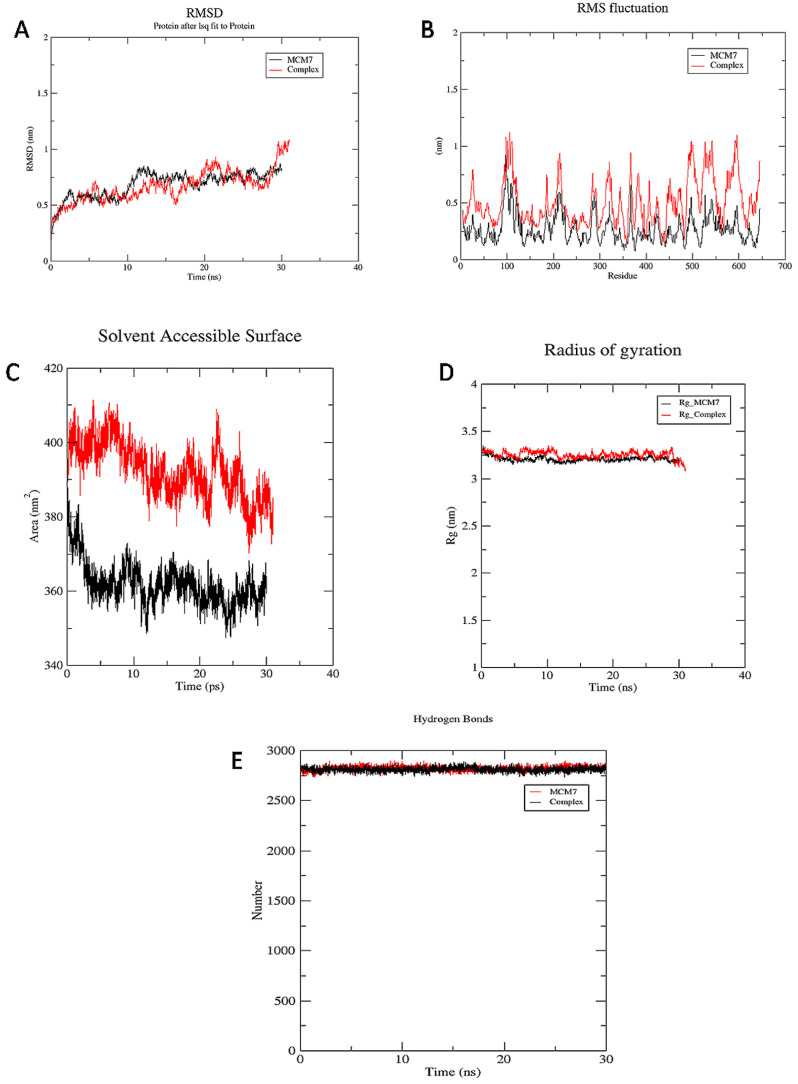
MD simulation studies of MCM7 (represented in black color) and the complex of MCM7 with UEFS99 (represented in red color). (**A**) RMSD, (**B**) RMSF, (**C**) SASA, (**D**) radius of gyration, (**E**) hydrogen bonds.

MD simulations of the protein and protein-ligand complex were carried out to illustrate the inhibition of MCM7 by the UEFS99. A comprehensive analysis of the dynamic trajectories which includes root mean square deviation (RMSD), RMS fluctuations (RMSFs), the radius of gyration (Rg), solvent accessible surface area (SASA), and H-bond profiling of MCM7 and its docked complex, retrieved through simulation was carried out. The analysis revealed very minutes differences between MCM7 and the complex. The RMSD values of the Ca atoms in MCM7 concerning the complex were reported across a 30 ns time frame, indicating a significant increase up to 1 ns, equilibrium at roughly 2.5 to 10 ns, followed by a small increase up to 30 ns, as shown in Figure 5**.**

#### 3.3.1. RMSD

The RMSD values of MCM7 and the complex are presented to show how the compounds affect structural stability and integrity. MCM7 structure revealed a marked increase in RMSD up to 2 ns in this plot and was subsequently optimized up to 27 ns at 0.7 nm, after which the RMSD of the complex reached 1 nm (Figure 5A). 

#### 3.3.2. RMSF

The comprehensive analysis of the RMSF profile of the MCM7 and the complex revealed variations of amino acids in the catalytic and non-catalytic regions. The catalytic site of MCM7 revealed fluctuations in the range of 0.2 to 0.3nm. However, the non-catalytic region in the complex structure has a high level of mobility, with fluctuations ranging from 0.2 to 1nm, as shown in Figure 5B. Highest fluctuations are observed in the regions of 360–380, 480–510, 524–530, and 570–610 residues. The residues Pro383, Gly384, Val385, Ala386, Lys387, Ser388, Gln389, Asp445, Glu446, Lys449, Ala487, and Asn489were observed to form close intra-molecular interactions with UEFS99 in the docking studies, are least fluctuated to form a stable complex.

#### 3.3.3. SASA

The solvent accessible surface area (SASA) of the complex and MCM7 structures were computed for 30 ns simulation. The SASA analysis revealed that the complex was exposed to the solvent region with an average area of 390 nm^2^. However, the SASA of MCM7 showed an average area of 360 nm^2^, as shown in Figure 5C.

#### 3.3.4. Rg

The radius of gyration (Rg) was measured for both structures. The radius of gyration computed for 30 ns illustrates the compactness of the protein with protein folding and unfolding by employing thermodynamic principles. Rg values for MCM7 protein and complex describe that both the structures maintain equilibrium with the average Rg value of 3.25 nm, as shown in Figure 5D. The findings suggest that Rg values for the complex were slightly increased from 7–12 ns. As a result, the binding of UEFS99’s with MCM7 altered the MCM7 protein’s microenvironment, causing conformational changes in the protein structure.

#### 3.3.5. Hydrogen Bond

Hydrogen bond occupancy and their number were calculated during 30 ns simulation. Intra-protein hydrogen bond occupancy within the MCM7 and the MCM7-UEFS99 complex remained stable throughout the study. Since the two graphs are overlapping, indicating high stability and good binding affinity of the ligand.

## 4. Conclusions

Multiple human cancers are caused by the overexpression of the DNA replication licensing complex component MCM7.The present article describes the biogenic compounds screening against MCM7 protein using the in silico art of technique to find promising MCM7 inhibitors. It is suggested that the hit compounds UEFS99, UEFS137, and UEFS428 show good binding with MCM7 protein, with UEFS99 also demonstrated stability as revealed by MD simulation. This study paves the wave for further wet lab validation (in vitro and in vivo study) of these compounds in the form of novel inhibitor development against MCM7 to fight cancers.

## Figures and Tables

**Table 1:** BE of hit compounds with MCM7 protein.

S. No.	Compounds	2D Structure	Binding Energy (kcal/mol)	Inhibition Constant (µM)	Interacting Residues
1	UEFS99	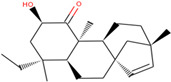	−9.95	2.49	Pro383, Gly384, Val385, Ala386, Lys387, Ser388, Gln389, Asp445, Glu446, Lys449, Ala487, and Asn489
2	UEFS137	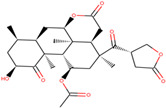	−8.92	5.13	Glu343, Ile344, Tyr345, Gly384, Val385, Ala386, Gln389, Leu533, Ile537, His536, Val540, and His541
3	UEFS428	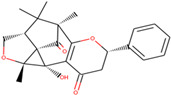	−8.71	9.53	Glu343, Ile344, Tyr345, His347, Gly384, Val385, Ala386, Gln389, Asp523, Leu533, His536, Ile537, and Val540
4	Ciprofloxacin *	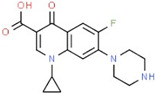	−6.50	52.27	Glu343, Ile344, Tyr345, Gly384, Val385, Ala386, Gln389, Asp523, Leu533, Ile537, and Val540

* Control compounds for MCM7 protein.

**Table 2:** Physicochemical and druglikenes properties of top 3 compounds.

Property	Model Name		Predicted Value		
			UEFS 428	UEFS 137	UEFS 99
			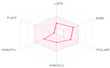	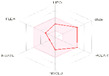	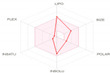
Physicochemical Properties	MW		380.43	504.57	316.48
	MR		100.3	125.97	94.45
	TPSA		72.83	133.27	37.3
Lipophilicity	iLOGP		0	1.6	2.99
	XLOGP3		1.02	1.59	5.67
	WLOGP		2.41	2.01	4.52
	MLOGP		1.56	1.73	4.05
	Silicos-IT Log P		4.04	2.87	4.3
	Consensus Log P		1.81	1.96	4.3
Estimated SOLubility (ESOL)	Log S		−2.93	−3.71	−5.31
	Solubility (mg/mL)		4.43 × 10^−1^	9.93 × 10^−2^	1.56 × 10^−3^
	Solubility (mol/L)		1.16 × 10^−3^	1.97 × 10^−4^	4.92 × 10^−6^
	Class		Soluble	Soluble	Moderately soluble
Pharmacokinetics	GI absorption		High	High	High
	BBB permeant		Yes	No	Yes
	Pgp substrate			Yes	No
	inhibitor	CYP1A2	No	No	No
		CYP2C19			
		CYP2C9			Yes
		CYP2D6			No
		CYP3A4			
	log Kp (cm/s)		−7.9	−8.25	−4.2
Druglikeness	Lipinski	Number of violations	0	1	0
	Ghose		0	2	0
	Veber		0	0	0
	Egan		0	1	0
	Muegge		0	0	1

**Table 3:** Toxicity prediction of top 3 compounds.

Classification	Target	UEFS 428		UEFS 137		UEFS 99	
		Prediction	Probability	Prediction	Probability	Prediction	Probability
Organ toxicity	Hepatotoxicity	-	0.75	-	0.85	-	0.71
Toxicity end points	Carcinogenicity	Active	0.51	-	0.61	-	0.67
	Immunotoxicity	-	0.96	Active	0.99	Active	0.97
	Mutagenicity	-	0.52	-	0.74	-	0.86
	Cytotoxicity	-	0.61	-	0.76	-	0.76
Tox21-Nuclear receptor signalling pathways	Aryl hydrocarbon Receptor	-	0.9	-	0.97	-	0.98
	Androgen Receptor	-	0.83	-	0.84	-	0.52
	Androgen Receptor Ligand Binding Domain	-	0.9	-	0.88	-	0.52
	Aromatase	-	0.84	-	0.84	-	0.96
	Estrogen Receptor Alpha	-	0.66	-	0.73	Active	0.53
	Estrogen Receptor Ligand Binding Domain	-	0.98	-	0.95	-	0.61
	PPAR-Gamma	-	0.93	-	0.92	-	0.98
Tox21-Stress response pathways	nrf2/ARE	-	0.87	-	0.98	-	0.91
	Heat shock factor response element	-	0.87	-	0.98	-	0.91
	Mitochondrial Membrane Potential	-	0.57	-	0.72	-	0.74
	p53	-	0.8	-	0.83	-	0.94
	ATPase family AAA domain-containing protein 5	-	0.9	-	0.91	-	0.96

## Data Availability

Not applicable.

## Supplementary Materials

The following are available online, Figure S1: BOILED-Egg estimation of top three compounds using SwissADME web tool, Figure S2: Interacting residues of MCM7 protein (PDB ID: 6XTX) with the attached ligand (phosphothiophosphoric acid-adenylate ester). Different color code represent the residual interaction types.
