# Supplementary material for: Computational Screening of Natural Compounds for Identification of Potential Anti-Cancer Agents Targeting MCM7 Protein
Source: Molecules. 2021 Sep 28;26(19):5878. doi: 10.3390/molecules26195878 (PMC8510405; doi:10.3390/molecules26195878)
Supplement: Supplementary file 1 [file molecules-26-05878-s001.zip › molecules-1351122-supplementary.pdf]

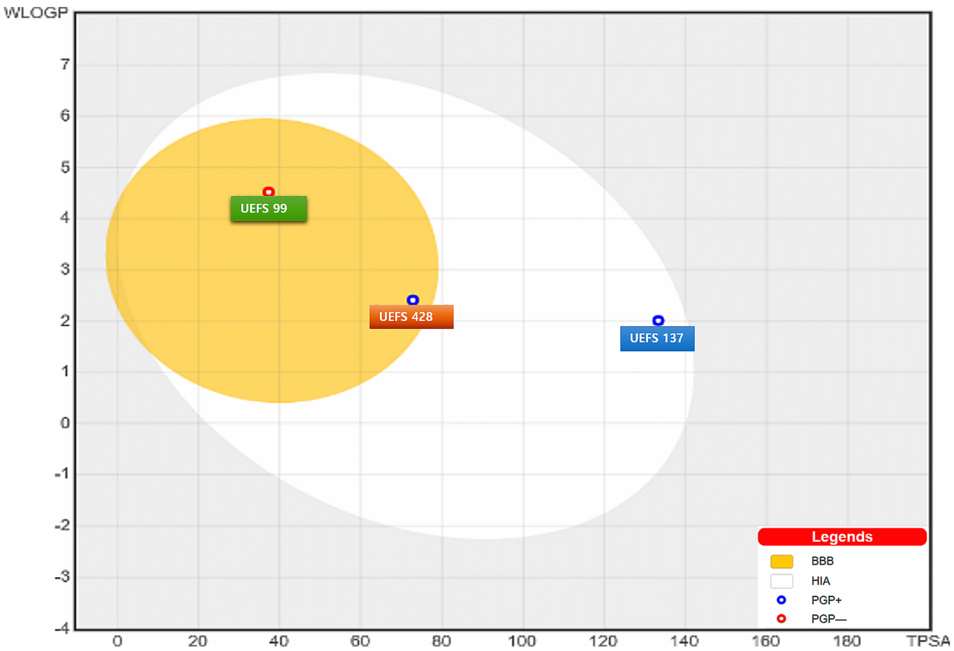

Supplementary Figure S1. BOILED-Egg estimation of top three compounds using SwissADME web tool.

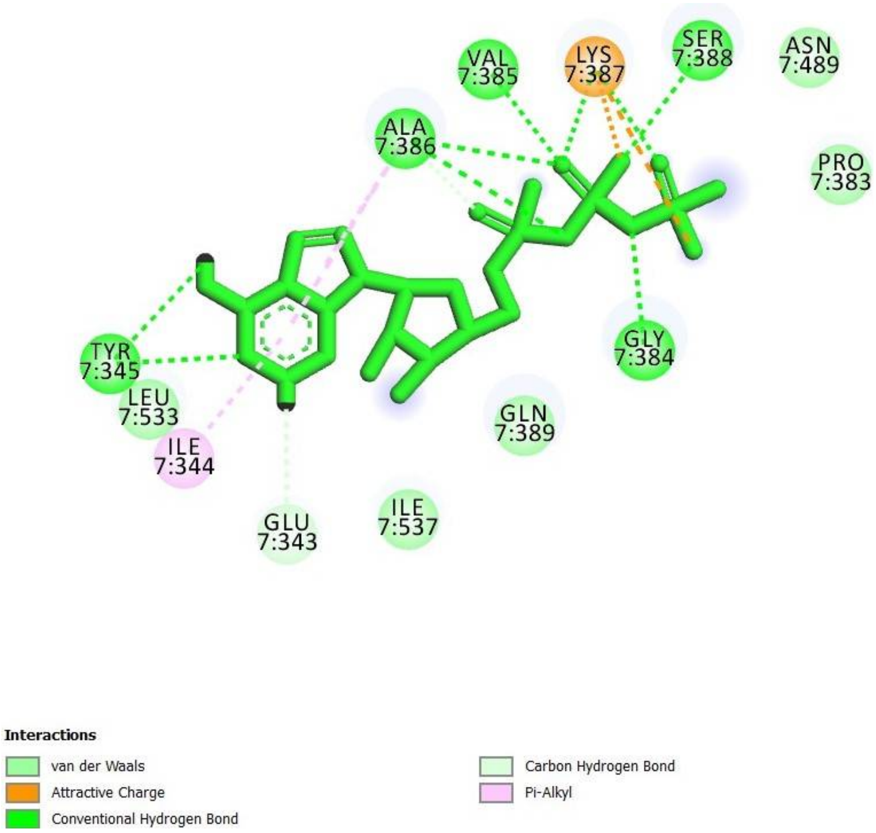

Supplementary Figure S2. Interacting residues of MCM7 protein (PDB ID: 6XTX) with the attached ligand (phosphothiophosphoric acid-adenylate ester). Different color code represent the residual interaction types.
